# Shared communication processes within healthcare teams for rare diseases and their influence on healthcare professionals' innovative behavior and patient satisfaction

**DOI:** 10.1186/1748-5908-6-40

**Published:** 2011-04-21

**Authors:** Henrike Hannemann-Weber, Maura Kessel, Karolina Budych, Carsten Schultz

**Affiliations:** 1 Institute for Technology and Innovation Management, Technische Universität Berlin, Strasse des 17. Juni 135, 10623 Berlin, Germany; 2 German Foundation for the chronically Ill, Fürth, Germany

## Abstract

**Background:**

A rare disease is a pattern of symptoms that afflicts less than five in 10,000 patients. However, as about 6,000 different rare disease patterns exist, they still have significant epidemiological relevance. We focus on rare diseases that affect multiple organs and thus demand that multidisciplinary healthcare professionals (HCPs) work together. In this context, standardized healthcare processes and concepts are mainly lacking, and a deficit of knowledge induces uncertainty and ambiguity. As such, individualized solutions for each patient are needed. This necessitates an intensive level of innovative individual behavior and thus, adequate idea generation. The final implementation of new healthcare concepts requires the integration of the expertise of all healthcare team members, including that of the patients. Therefore, knowledge sharing between HCPs and shared decision making between HCPs and patients are important. The objective of this study is to assess the contribution of shared communication and decision-making processes in patient-centered healthcare teams to the generation of innovative concepts and consequently to improvements in patient satisfaction.

**Methods:**

A theoretical framework covering interaction processes and explorative outcomes, and using patient satisfaction as a measure for operational performance, was developed based on healthcare management, innovation, and social science literature. This theoretical framework forms the basis for a three-phase, mixed-method study. Exploratory phase I will first involve collecting qualitative data to detect central interaction barriers within healthcare teams. The results are related back to theory, and testable hypotheses will be derived. Phase II then comprises the testing of hypotheses through a quantitative survey of patients and their HCPs in six different rare disease patterns. For each of the six diseases, the sample should comprise an average of 30 patients with six HCP per patient-centered healthcare team. Finally, in phase III, qualitative data will be generated via semi-structured telephone interviews with patients to gain a deeper understanding of the communication processes and initiatives that generate innovative solutions.

**Discussion:**

The findings of this proposed study will help to elucidate the necessity of individualized innovative solutions for patients with rare diseases. Therefore, this study will pinpoint the primary interaction and communication processes in multidisciplinary teams, as well as the required interplay between exploratory outcomes and operational performance. Hence, this study will provide healthcare institutions and HCPs with results and information essential for elaborating and implementing individual care solutions through the establishment of appropriate interaction and communication structures and processes within patient-centered healthcare teams.

## Background

**Figure 1:**
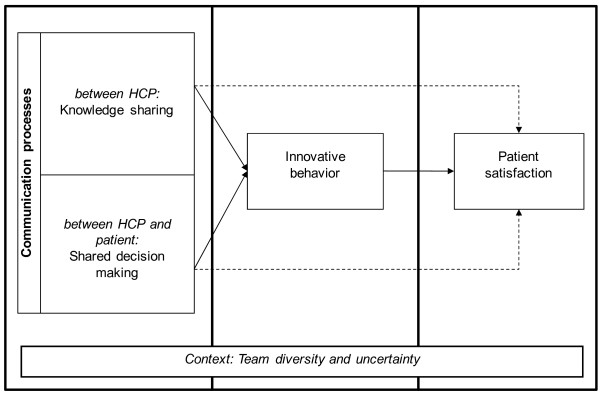
**Study framework**.

Rare diseases are defined as specific disease patterns with a prevalence of less than five in 10,000 [1] patients. This infrequent prevalence causes a serious deficit of expert knowledge that often induces uncertainty, ambiguity, and unpredictability in routine care. However, patients with rare diseases frequently have a strong need for complex and multidisciplinary treatment. Expertise and knowledge are required, but they are often located in dispersed centers of expertise, and are thus disconnected from the local healthcare environment of patients. Standardized healthcare guidelines are lacking due to the great variance of symptoms and treatment processes within each disease pattern. Therefore, multidisciplinary healthcare teams, diverse in education and function, are tasked with creating new, individual, patient-centered solutions to improving patients' long-term healthcare situation. We define this necessary innovative behavior of healthcare providers (HCPs) as the intensity of proactive behavior and improvisation to find adequate individualized solutions for each patient and to implement new processes, products, or procedures to enhance medical outcomes. In addition to the emerging incremental adaptations of current healthcare processes, initiatives and new solutions for medical products and procedures arise that have to be transferred to other HCPs. To cope with the complexity of rare diseases, idea generation and implementation both require the integration all team members' expertise, including that of the patient. As such, communication processes between the involved actors play an essential role. Our study focuses on two different communication processes, knowledge sharing between HCPs and shared decision making between HCPs and patients. Based on two different literature streams, innovation management and health service research, we suggest that both communication processes will foster HCPs' innovative behavior, which in turn influences patient satisfaction positively (see Figure 1). These communication processes are influenced by specific characteristics of rare diseases. In particular, HCPs and patients have to deal with the high functional diversity of the team [2-4] and high environmental uncertainty that affect routine and explorative processes [5,6]. In this study, we develop a theoretical framework and derive hypotheses, as indicated in the study framework above. We also describe the study plan and discuss central contributions of this study.

In this study, we develop a theoretical framework and derive hypotheses, as indicated in the study framework above. We also describe the study plan and discuss central contributions of this study.

### Knowledge sharing and its influence on innovative behavior and patient satisfaction

We define innovative behavior as the introduction and implementation of new ideas, processes, products, or procedures designed to significantly benefit the patient. Several authors see knowledge as a critical resource of organizations, networks, or teams that provides a sustainable advantage for innovative performance outcomes [7-9]. This assertion is applicable to knowledge-intense working contexts where information is broadly lacking - the treatment of patients with rare diseases. Knowledge, defined as 'a fluid mix of framed experience, values, contextual information, and expert insights [...]' [8], represents the basis for evaluating and incorporating new experiences and information to create new healthcare concepts and treatments fitting patients' needs [8]. Different HCPs carry different expertise. Therefore, diverse teams possess a broader range of explicit knowledge and a larger pool of abilities and skills, and thereby may lead to improved patient outcomes [2,10]. The variety of knowledge carriers underlies the importance of knowledge-sharing processes between members of healthcare teams. If knowledge is not shared, cognitive resources available within a team remain idle [11]. Strong relationships and interactive knowledge sharing enable the team to create new solutions [12,13] by combining new with existing knowledge to come up with novel ideas and concepts [14]. In our study, knowledge sharing is considered to be an interactive communication process between at least two HCPs. It is characterized by various communication attributes, such as the frequency and reciprocity of knowledge exchange, the multiplicity of knowledge content [15], and the quality and strength of the HCPs' relationships [16]. Referring to healthcare teams dealing with patients with rare diseases, we assume that internal knowledge-sharing processes start immediately after a multidisciplinary healthcare team is assembled. This builds a foundation for essential innovative healthcare activities. The meta-analytic overview from van Wijk [17] supports this idea by showing a significant overall correlation between knowledge sharing and innovative performance, and this correlation underlines our assumption that within healthcare teams, interactive knowledge-sharing processes positively influence HCPs' innovative behavior.

In addition to the need for knowledge sharing for explorative outcomes, operational performance also depends on the intensity of knowledge sharing between HCPs, particularly specific knowledge related to more routine activities [17]. Knowledge sharing can also be seen as an essential aspect of meeting patients' needs in the operational treatment of daily healthcare processes. As such, we suggest that intensive information exchange concerning the care of patients with rare diseases significantly affects patient satisfaction by better fitting their permanent needs.

### Shared decision making and its influence on innovative behavior and patient satisfaction

Although the concept of knowledge sharing focuses mainly on HCPs, the interaction with the patient, and in particular the process of shared decision making (SDM), must also be addressed. SDM can be defined as an interactive process in which at least two participants - physician and patient - share information and equally reach an agreement on the treatment to implement [18,19]. Despite the considerable challenges associated with decision making for rare diseases, investigations into the shared decision-making process, its implications, and its impact on innovative behavior in the setting of rare diseases have been lacking. Moreover, outside of the healthcare context, researchers have typically studied participation effects in the organizational context, focusing for example on the leadership style and its impact on employees' innovativeness [20]. The influence of the patient's participation in decision making on the service provider's innovative behavior has received minimal attention in the literature to date. Preliminary indications have arisen from the literature review and Delphi study by Fleuren [21]. They identified patient cooperation as a relevant determinant of innovative behavior within healthcare organizations. Especially in the context of rare diseases characterized by uncertainty due to insufficient knowledge, mutual willingness to influence and to be influenced is essential for the development of creative ideas and their transformation into workable methods, products, and services. We argue that as the patient becomes more involved in the decision-making process, the solutions developed by HCPs may be re-examined and re-evaluated [22]. Hence, it enables HCPs to critically process their own creative ideas and to pursue those that will best meet the patients' expectations and requirements. We therefore state that there is solid justification for exploring participation and particularly shared decision making as an important determinant of innovative behavior of HCPs. Additionally, through fostering a common understanding of the disease between patient and HCPs, patient involvement in treatment decisions may help the HCPs to better meet the patient's needs by providing customized healthcare [23]. The gap between the patient's expectations and their perception of performance will diminish [24]. Thus, shared decision making also has a positive effect on patient satisfaction.

### Innovative behavior and its influence on patient satisfaction

New medical products and processes require innovative behavior from HCPs. This is of particular importance for patients with rare diseases, because innovative concepts must compensate for limited knowledge and missing routines. As a result, the healthcare team improves its ability to serve and help patients [25]; the patient will receive appropriate and highly suitable help, and will be more satisfied. As such, we suggest that innovative behavior positively relates to overall healthcare performance and more specifically to patient satisfaction.

In conclusion, based on the above-mentioned assumptions, this study aims to test the following hypotheses concerning the impact on patient satisfaction of knowledge sharing and shared decision making mediated by innovative behavior of individual HCPs operating under uncertain conditions in multidisciplinary teams:

Hypothesis 1: Knowledge sharing between HCPs in patient-centered teams positively influences innovative behavior.

Hypothesis 2: Knowledge sharing between HCPs in patient-centered teams has a direct positive influence on patient satisfaction.

Hypothesis 3: Patient involvement in shared decision making positively influences HCPs' innovative behavior.

Hypothesis 4: Patient involvement in shared decision making has a direct positive influence on patient satisfaction.

Hypothesis 5: HCPs' innovative behavior positively influences patient satisfaction.

## Methods

### Design

The overall design is an empirical study in which a series of attributes of individuals and teams are measured to test the developed hypotheses. A three-phase, mixed-method and multi-level study will be conducted. Phase I is an exploratory study, phase II is the quantitative part of the main study, and phase III is the qualitative part of the main study.

### Participants and sample size

Through expert interviews with various physicians specializing in the care of rare diseases and with representatives of self-help organizations, we assessed a wide range of disease patterns and finally focused the study on six different rare diseases. They were selected by pre-defined criteria: a requirement for multidisciplinary team work, regionally dispersed expertise, limited experience, a degree of uncertainty due to an absence of knowledge and routines, and extraordinary individual healthcare demands. We tried to choose diseases that mainly differ in care intensity, level of suffering, patients' age of disease outbreak (adults versus children), affected organs, and prevalence. Thus, in an iterative process, we finally chose the following diseases to test our theoretical framework: Amyotrophic lateral sclerosis, Marfan's syndrome, Wilson's disease, Epidermolysis bullosa, Duchenne muscular dystrophy, and Neurodegeneration with brain iron accumulation.

Patients will be recruited via brochures placed in centers of expertise and specialized hospitals for rare diseases as well as in non-profit self-help organizations. For each of the six diseases, the sample should comprise 30 patients. Only patients and their HCPs whose permanent residence is in Germany will be recruited. To shed light on shared communication processes among healthcare teams, we will address several HCPs of each patient-centered healthcare team. Patients who have declared their participation will then be asked to return a list indicating all members of their healthcare team. On average, we expect six HCPs per patient-centered healthcare team, *e.g.*, general practitioners, nurses, heath care aides, physicians in hospitals or ambulatory settings, and various therapists and social workers involved in operational healthcare processes. Out of our chosen diseases, neurodegeneration with brain iron accumulation has the smallest prevalence, with about 50 patients in Germany. To ensure comparability we will send out 50 patients' questionnaires for all the selected diseases and expect a response rate of 60%. We anticipate that a high number of patients will participate in our study because they typically display a high level of personal concern. Moreover, our exploratory pre-study in phase I indicated that both patients and HCPs were enthusiastic to participate. Therefore, we also expect a relatively high response rate of 40% for the six HCPs per team. In total, we expect to build on data from 180 patients and 432 HCPs.

### Data collection

#### Phase I: exploratory pre-study

In an initial pre-study, we collected data via exploratory interviews to detect central barriers teams have to cope with in their daily work with patients suffering from rare diseases. We collected data from four patient-centered healthcare teams, including four patients and relatives together with 16 HCPs such as nurses, healthcare givers, doctors, therapists, health insurance agents, and service employees of medical device producers. In addition to resource restrictions, we mainly detected limitations in communication processes between HCPs and patients as well as between members of healthcare teams. Therefore, our findings highlighted a significant need for specific intra-team processes such as extensive knowledge sharing and shared decision making within healthcare teams including patients. Additionally, the interviews confirmed the relevance of individualized solutions to improving long-term healthcare and consequently to increasing patient satisfaction.

#### Phase II: quantitative main study

The main study is a deductive analysis aiming to test our hypotheses mentioned above - that knowledge sharing and shared decision making positively influence HCPs' innovative behavior, which consequently leads to better patient satisfaction. Questionnaires will be sent out to our above-described sample evaluating demographic data, frequency, reciprocity and multiplexity of knowledge sharing, the role of shared decision making between patients and HCPs, individual innovative behavior, and patient satisfaction. Together with the questionnaire, each patient will be asked to return a list indicating their healthcare team members. In a second step, we will send a questionnaire to each of the stated healthcare team members evaluating demographic data, functional diversity, environmental uncertainty, frequency, reciprocity and multiplexity of knowledge sharing, and individual innovative behavior.

#### Phase III: qualitative main study

After receiving the questionnaires, we will conduct semi-structured telephone interviews with the patients. The interviews will last approximately 20 minutes and will be designed in accordance with recommendations for qualitative research [26-28]. The objective of these interviews is to gain a deeper understanding of the processes of knowledge sharing and shared decision making among healthcare team members and their initiatives to find innovative solutions. By combining our qualitative and quantitative results, we aim to formulate concrete proposals on how to optimize communication and innovation processes for rare diseases.

### Measurement and analysis

All questionnaire items will be rated on a seven-point Likert scale ranging from 1 'strongly disagree' to 7 'strongly agree.' In line with our study framework, we will examine the following four concepts: knowledge sharing, shared decision making, HCPs' innovative behavior, and patient satisfaction. To examine knowledge sharing within healthcare teams, every participant will be asked to indicate how often (daily, weekly, monthly, or less than once a month) he/she interacts with each team member to exchange procedural knowledge (*e.g.*, information about healthcare procedures and processes) and declarative knowledge (*e.g. *information about diagnosis, symptoms, or therapies). This means of measuring knowledge sharing was adapted from Bakker *et al. *[15] and will result in a matrix that captures the intensity of knowledge sharing regarding procedural and declarative information between members of each team. We will use the nine-item Shared Decision-Making Questionnaire (SDM-Q-9) from Kriston *et al. *[19] to assess the use of shared decision making within healthcare teams. SDM is defined here as an interactive process in which patients and their HCPs share information equally in reaching an agreement on treatment. Hence, the questionnaire consists of nine items each describing one step of the SDM process. A sample item is 'My doctor helped me understand all the information.' Innovative behavior will be measured with a scale combined from two previously developed scales: the creativity scale of Zhou and George [29] (three items, *e.g.*, 'I am/He/She is a good source of creative ideas') and the innovation scale developed by Scott and Bruce [20] (two items, *e.g.*, 'I/He/She promote(s) and champion(s) ideas to others.'). We chose this combination of items because they represent the major stages in the individual innovative behavior process (problem identification, information searching and encoding, idea generation, and implementation) and because they are the most appropriate for the given context of healthcare teams working on uncertain tasks such as rare diseases. The innovative behavior of each HCP will be measured using a two-informant design via self-evaluation and external evaluation through patients. To explore patient satisfaction, we will use a patient satisfaction scale based on the Munich Patient Satisfaction Scale (MPSS-24), which in its original form consists of 24 items mainly addressing socio-emotional and communicative aspects of the patient-HCP relationship [30]. For this study, we omitted six items, *e.g.*, 'The doctors are being interested in my problems;' additionally, we included an item to measure overall satisfaction. We chose the MPSS-24 because it focuses on the HCPs' competence. The scale will be adopted for each subgroup (doctors, physicians, healthcare givers, therapists). In addition, patients also rated their overall level of satisfaction with healthcare on a 10-point scale ranging from 1 (least satisfied) to 10 (most satisfied). In addition, we will control for several aspects to limit the influence of unobserved variance. We will control for functional diversity among healthcare teams by drawing on past research [2,31] that operationalizes this concept by addressing the tenure, educational background, and functional background of the team. In line with recommendations on how to measure diversity [32], we will measure the mentioned variables using Blau's index of heterogeneity, 1- ∑p_i_^2 ^[33]. In this formula, p represents the proportion of a team in the respective diversity category, and i is the number of different categories represented within a team. Thus, an index of 0 indicates no diversity, while a higher index score indicates that more diversity exists in the measured variable among team members. Additionally, we integrate the context of uncertainty as a second control variable, which will be measured by a three-item scale originally used by Gladys *et al.*, *e.g.*, 'The intensity of the patients' healthcare is unpredictable' [34]. The statistical analysis will explore the relationships between the two predictor variables (knowledge sharing and shared decision making) and both the dependent variable of patient satisfaction and the mediating effect of HCPs' innovative behavior by controlling for functional diversity within each team and environmental uncertainty. The theoretical model will be tested using multiple regression analysis and structural equation modeling. In addition to phase II, we will evaluate the qualitative data within phase III using MAXQDA in line with recommendations for qualitative research and grounded theory [26-28].

### Ethical considerations

Ethics approval for the project was received from the Research Ethics Board of Technische Universität Berlin, Institut für Psychologie und Arbeitswissenschaft (approved 08 December 2010; ethics number: SC_01_20101116).

## Discussion

Patients with rare diseases regularly encounter serious deficits in HCPs' expertise and in treatment guidelines, and this causes a high level of uncertainty and ambiguity in routine healthcare processes. In this study, we argue that the assembly of multidisciplinary healthcare teams consisting of both routine and specialized HCPs is required to generate individually tailored healthcare concepts. Team diversity, *i.e.*, the amount of multidisciplinarity and the level of qualification within a healthcare team, is considered to be a key contextual element. Moreover, uncertainty and unpredictability create an inability to predict accurately what the outcomes of decisions might be [5,6]. This leads to an unstable and uncontrolled situation for the patient [35,36]. In line with these specific conditions, the proposed theory-based approach will shed light on interaction processes from an integrated perspective. After identifying the main theoretical communication processes within healthcare teams, they will be empirically tested. Our study will investigate patients' needs via qualitative data and their satisfaction with the healthcare situation via quantitative data. Moreover, HCPs' innovative behavior will be investigated with special attention to their communication activities within teams and with the patient. This allows us to consider healthcare teams as a whole, integrating the patients in particular. Thus, healthcare teams include the whole multidisciplinary set of HCPs including relatives and patients. Referring to our study framework, healthcare teams with norms for shared decision making and intensive knowledge sharing that facilitate open communication among team members may encourage individuals to innovate, which in turn increases individual patient satisfaction. Hence, this study will provide unique information on the most important factors for improving the long-term care of patients with rare diseases through the development of individual innovative care concepts. We anticipate that our results will significantly contribute to research by analyzing the role of knowledge sharing and shared decision making within patient-centered healthcare teams, and their impact on HCPs' innovative attempts to better meet patient's needs and thereby improve patient satisfaction. Supported by the qualitative results, we aim to provide practical solutions: implementing and subsequently institutionalizing central shared communications processes within healthcare teams including the patient may be key in promoting patient-centered, individualized innovative concepts for patients with rare diseases. Our results will provide healthcare institutions and HCPs with essential information for elaborating and implementing individual care solutions through the establishment of appropriate interaction and communication structures and processes. With respect to the limitations of a single country study, we suggest that future studies expand this German sample to an international sample to generalize the results and to dissociate them from country-specific confounding variables.

## Competing interests

The authors declare that they have no competing interests.

## Authors' contributions

HH-W, MK, KB and CS conceived and developed the study. HH-W and MK drafted the study protocol and lead and coordinate the study under the supervision of CS. KB and CS helped to draft this study protocol. HH-W, MK, KB and CS developed the questionnaires and interview guidelines; HH-W, MK and KB are responsible for the data collection. CS prepared the ethical approval document. All authors read, and approved the final manuscript. CS is its guarantor.
